# History of large-for-gestational-age birth is independently associated with subsequent gestational diabetes in Chinese multiparous women: a retrospective cohort study

**DOI:** 10.3389/fendo.2025.1678319

**Published:** 2025-12-04

**Authors:** Juan Yang, Shilin Zhong, Min Wang, Yuzhen Liu, Ying Wang, Yuqing Deng, Kaidong Ma, Ao Yang, Yanmei Li, Chang Xu

**Affiliations:** 1 Center of Obstetrics and Gynecology, Peking University Shenzhen Hospital, Shenzhen, Guangdong, China; 2 Institute of Obstetrics and Gynecology, Shenzhen Peking University-Hong Kong University of Science and Technology (PKU-HKUST) Medical Center, Shenzhen, Guangdong, China; 3 Peking University Shenzhen Hospital, Shenzhen Key Laboratory on Technology for Early Diagnosis of Major Gynecologic Diseases, Shenzhen, Guangdong, China; 4 Department of Obstetrics and Gynecology, Guangzhou Women and Children’s Medical Center, Guangzhou Medical University, Guangzhou, Guangdong, China; 5 Intelligent Hospital Research Academy, Peking University Shenzhen Hospital, Shenzhen, Guangdong, China

**Keywords:** gestational diabetes mellitus, large-for-gestational-age, macrosomia, birth weight, risk

## Abstract

**Background:**

While prior macrosomia has been linked to subsequent gestational diabetes mellitus (GDM), the association between a history of large-for-gestational-age (LGA) births and subsequent GDM, as well as its comparative relevance versus macrosomia, remain underexplored in Chinese populations, highlighting a critical gap in perinatal risk stratification.

**Aim:**

To determine whether a history of LGA (f-LGA) birth is independently associated with subsequent GDM (s-GDM) and compare its associative relevance versus macrosomia in Chinese multiparous women.

**Method:**

This retrospective cohort study included women with consecutive singleton deliveries (2002-2024) at Peking University Shenzhen Hospital. Exposures were LGA (birth weight ≥90th percentile, China 2020 standards) or macrosomia (≥4000 g) in the first pregnancy; outcome was s-GDM (IADPSG criteria). Associations were evaluated using restricted cubic splines (RCS) and multivariable logistic regression with sequential adjustment (unadjusted, minimally adjusted, and fully adjusted). Stratified analyses assessed effect modification by prior GDM, maternal age, and pre-pregnancy BMI in the first pregnancy.

**Results:**

Among 3,110 women analyzed, RCS analyses revealed a significant monotonic increase in s-GDM risk with higher birth weight percentiles (P for association < 0.001, P for nonlinearity = 0.010 after full adjustment). Although absolute birth weight categories showed a statistically significant overall association (P for association = 0.033), no dose-response pattern was observed. After full adjustment, prior LGA remained significantly associated with s-GDM (aOR = 1.458, 95% CI: 1.045–2.035), whereas prior macrosomia did not (aOR = 1.271, 95% CI: 0.782–2.067). Significant positive trends existed across percentile categories (*P* for trend = 0.025) but not absolute weight categories (*P* for trend = 0.310). Stratified analyses demonstrated robust associations of f-LGA with s-GDM specifically in women without prior GDM (aOR = 1.544, 95% CI: 1.069–2.232), aged <35 years (aOR=1.668, 95% CI: 1.095–2.542), and with normal pre-pregnancy BMI (aOR=1.757, 95% CI: 1.169–2.641) (*P* for trend < 0.001 for all strata).

**Conclusion:**

A history of LGA birth is independently associated with s-GDM risk in Chinese multiparous women, particularly among those without prior dysglycemia, aged <35 years, or with normal BMI. The percentile-defined LGA definition demonstrates superior associative relevance compared to absolute macrosomia, supporting its application in precision screening for multiparous pregnancies.

## 1 Introduction

Gestational diabetes mellitus (GDM), characterized by the onset of glucose intolerance during pregnancy in previously normoglycemic individuals, exhibits a globally heterogeneous prevalence ranging from 2% to 25% (1). In China, the incidence of GDM is 14.8% according to IADPSG criteria (2). This condition significantly increases perinatal risks, including preterm delivery (3), hypertensive disorders (4), emergent cesarean section (5), and neonatal complications (6–10), while also raising long-term mother-offspring sequelae, notably maternal progression to type 2 diabetes (T2DM) (11), and cardiometabolic vulnerability in offspring that persists into adolescence and adulthood (11, 12).

While established GDM risk factors encompass advanced maternal age (13), obesity (14), polycystic ovary syndrome (15), familial diabetes (16), and prior GDM (17), emerging evidence implicates previous macrosomia (birth weight ≥4,000 g) as a significant predictor. Meta-analyses identify prior macrosomia as a high-risk factor for recurrent GDM (17, 18), potentially mediated by persistent maternal metabolic dysfunction including insulin resistance (19), dyslipidemia (20), and weight dysregulation (21, 22).

Large-for-gestational-age (LGA) infants, which exceed the 90th percentile for sex-specific gestational age, represent a distinct fetal overgrowth phenotype. In China, the prevalence of LGA ranges nationally from 7% to 22% (23). Though sharing risk factors such as maternal obesity (24) and complications including birth trauma (25) with macrosomia, LGA demonstrates unique attributes: higher population frequency (26), occurrence in preterm infants, stronger genetic determinants (27), and association with maternal metabolic dysregulation potentially predisposing to subsequent GDM (28).Crucially, women with recurrent GDM exhibit significantly higher prior birth weight centiles than non-recurrent counterparts (29), suggesting LGA may reflect enduring metabolic perturbations.

Current literature exhibits critical knowledge gaps regarding the association between LGA and subsequent gestational diabetes risk. First, the sole study investigating a history of LGA/macrosomia as factors associated with subsequent GDM, which was conducted in an Israeli cohort, excluded women with prior GDM (30), thereby limiting generalizability to high-risk populations. Second, existing research on prior LGA delivery predominantly centers on postpartum metabolic sequelae (31) and long-term maternal T2DM risk (32), with insufficient attention to its immediate implications for subsequent pregnancy glycemic dysregulation. Third, while multiple international studies demonstrate macrosomia as a significant risk factor for recurrent GDM (17, 33), Chinese investigations report contradictory null associations (34), creating population-specific uncertainty.

Consequently, the associative validity of two distinct types of birth weight classification systems, LGA (percentile-defined overgrowth) versus macrosomia (absolute overgrowth), in relation to subsequent gestational diabetes risk remains unestablished in Chinese populations. Therefore, this retrospective cohort study aims to: (1) determine whether a history of LGA is independently associated with incident GDM in Chinese multiparous women, (2) comparatively evaluate the associative relevance of LGA versus macrosomia classifications, and (3) assess effect modification by maternal age, pre-pregnancy BMI, and prior GDM status. These findings will inform precision screening protocols for high-risk pregnancies, addressing a critical evidence gap in perinatal risk stratification.

## 2 Methods

### 2.1 Study design and participants

This study utilized the same cohort (January 2002 to March 2024) as our prior investigation into the impact of GDM history on LGA infants and macrosomia in subsequent pregnancies (35). For the present analysis, which focuses on the association between a history of LGA birth and subsequent GDM, we further excluded individuals with additional complications in the first pregnancy. Eligibility criteria required a gestational age at delivery ≥28 weeks, maternal age between 18 and 50 years, and complete medical records for both pregnancies. Exclusion criteria included: stillbirth or fetal malformation; multiple pregnancies; pregestational diabetes mellitus in either pregnancy; presence of other complications in the second pregnancy (chronic hypertension, preeclampsia, intrahepatic cholestasis, or severe cardiac/renal disease); and cases lacking data on GDM diagnosis, pre-pregnancy BMI, gestational weight gain, or newborn birth weight. Due to the study’s long time span, some early cases were excluded due to missing critical variables (including pre-pregnancy BMI, gestational weight gain, and GDM diagnostic information), which were necessary for the analyses. The study protocol was approved by the Research Ethics Committee of Peking University Shenzhen Hospital (Approval No: #2023-103-R1), and informed consent was waived due to the retrospective design.

### 2.2 Definitions and diagnostic criteria

GDM diagnostic protocols at our center evolved across the 2002–2024 study period but were harmonized to ensure analytical consistency for outcome ascertainment: Before 2010, a two-step method was used—all pregnant women first completed a 50g glucose challenge test (GCT), and those with a 1-hour glucose level ≥7.8 mmol/L (screening-positive) underwent a 100g oral glucose tolerance test (100g-OGTT) measuring glucose at 0h (fasting), 1h, 2h, and 3h, with GDM diagnosed if two or more thresholds were met (fasting ≥5.8 mmol/L, 1h ≥10.6 mmol/L, 2h ≥9.2 mmol/L, 3h ≥8.1 mmol/L); after 2010, the International Association of Diabetes and Pregnancy Study Groups (IADPSG) (36) one-step method was adopted, where all pregnant women directly completed a 75g oral glucose tolerance test (75g-OGTT) at 24–28 weeks, with GDM diagnosed if any one threshold was exceeded (fasting ≥5.1 mmol/L, 1h ≥10.0 mmol/L, 2h ≥8.5 mmol/L). Macrosomia (MAC) referred to birth weight ≥4000 g. LGA infants were defined as birth weight exceeding the 90th percentile for gestational age and sex, based on the 2020 Chinese growth reference standards (37). Birth weight in the first pregnancy (f-BW) was categorized using two distinct systems: i) Absolute categories: C1 (<2500 g), C2 (2500–2999 g), C3 (3000–3499 g; reference group), C4 (3500–3999 g), C5 (≥4000 g; MAC). ii) Percentile categories: P1 (<3^rd^ percentile), P2 (3^rd^–<10^th^ percentile), P3 (10^th^–<25^th^ percentile), P4 (25^th^–<50^th^ percentile; reference group), P5 (50^th^–<75^th^ percentile), P6 (75^th^–<90^th^ percentile), P7 (90^th^–<97^th^ percentile), P8 (≥97^th^ percentile) (37). LGA comprised infants in categories P7 and P8 (≥90^th^ percentile). The interpregnancy interval (IPI) was calculated as the interval in months between the delivery date of the first pregnancy and the estimated conception date of the second pregnancy (38). Interpregnancy weight change (IPWC) was computed as the difference between pre-pregnancy body mass index (BMI) in the second pregnancy and pre-pregnancy BMI in the first pregnancy (39). Pre-pregnancy BMI was categorized as: underweight (UW, <18.5 kg/m²), normal weight (NW, 18.5–24.0 kg/m²), or overweight/obesity (OB, ≥24.0 kg/m²).

### 2.3 Data collection

Demographic, obstetric, and neonatal data were extracted from electronic medical records and the maternal-fetal health registry. Variables included maternal age, pre-pregnancy BMI, GWG, delivery mode, gestational age, neonatal sex, birth weight, and comorbidities, including GDM, intrahepatic cholestasis of pregnancy and hypertensive disorders of pregnancy. Data from the first (f-) and second (s-) pregnancies were matched for each participant to create a longitudinal dataset.

### 2.4 Statistical analysis

Continuous variables were summarized as mean ± standard deviation (SD) for normally distributed data or median (interquartile range, IQR) for non-normally distributed data; categorical variables were presented as frequencies (%). Group comparisons utilized independent t-tests, Mann-Whitney U tests, or Chi-square tests, as appropriate for variable type and distribution. The analysis proceeded sequentially through three phases. First, we performed restricted cubic spline (RCS) analyses with three knots (at the 10th, 50th, and 90th percentiles) (40) to evaluate potential non-linear associations between first-pregnancy birth weight (f-BW) and the risk of subsequent gestational diabetes mellitus (s-GDM) from two perspectives: (1) f-BW as a continuous variable, and (2) f-BW categorized by percentiles (P1-P8); linear trends across these ordinal categories were assessed using multivariable logistic regression. Second, the associations between extreme exposure categories in the first pregnancy, namely macrosomia (f-MAC, C5) and large-for-gestational-age (f-LGA, combining P7 and P8), and s-GDM were evaluated using three nested multivariable logistic regression models: Model 1 (unadjusted); Model 2 (minimally adjusted), which included covariates identified as potential confounders by a >10% change in the odds ratio (OR) for the f-LGA/f-MAC and s-GDM association when added to Model 1 (s-BMI); and Model 3 (fully adjusted), which built on Model 2 by adding variables with P < 0.1 in univariate analyses of associations with s-GDM (f-GDM, f-CS, f-GWD, IPI, IPWC, s-BMI, s-MA, and s-GWG). Third, stratified analyses assessed potential effect modification by prior GDM status (no f-GDM [f-ND]/f-GDM), maternal age (<35 years [s-YMA]/≥35 years [s-AMA]), and pre-pregnancy BMI (underweight [s-UW]/normal weight [s-NW]/overweight or obesity [s-OB]), with formal tests for heterogeneity conducted using interaction terms within multivariable logistic regression models. Statistical significance was defined as a two-sided P value < 0.05. All analyses were performed using SPSS version 26.0 (IBM Corp.) and R version 4.2.0 (R Foundation for Statistical Computing).

## 3 Results

### 3.1 Comparison of baseline characteristics between the first and second pregnancies

**Figure 1:**
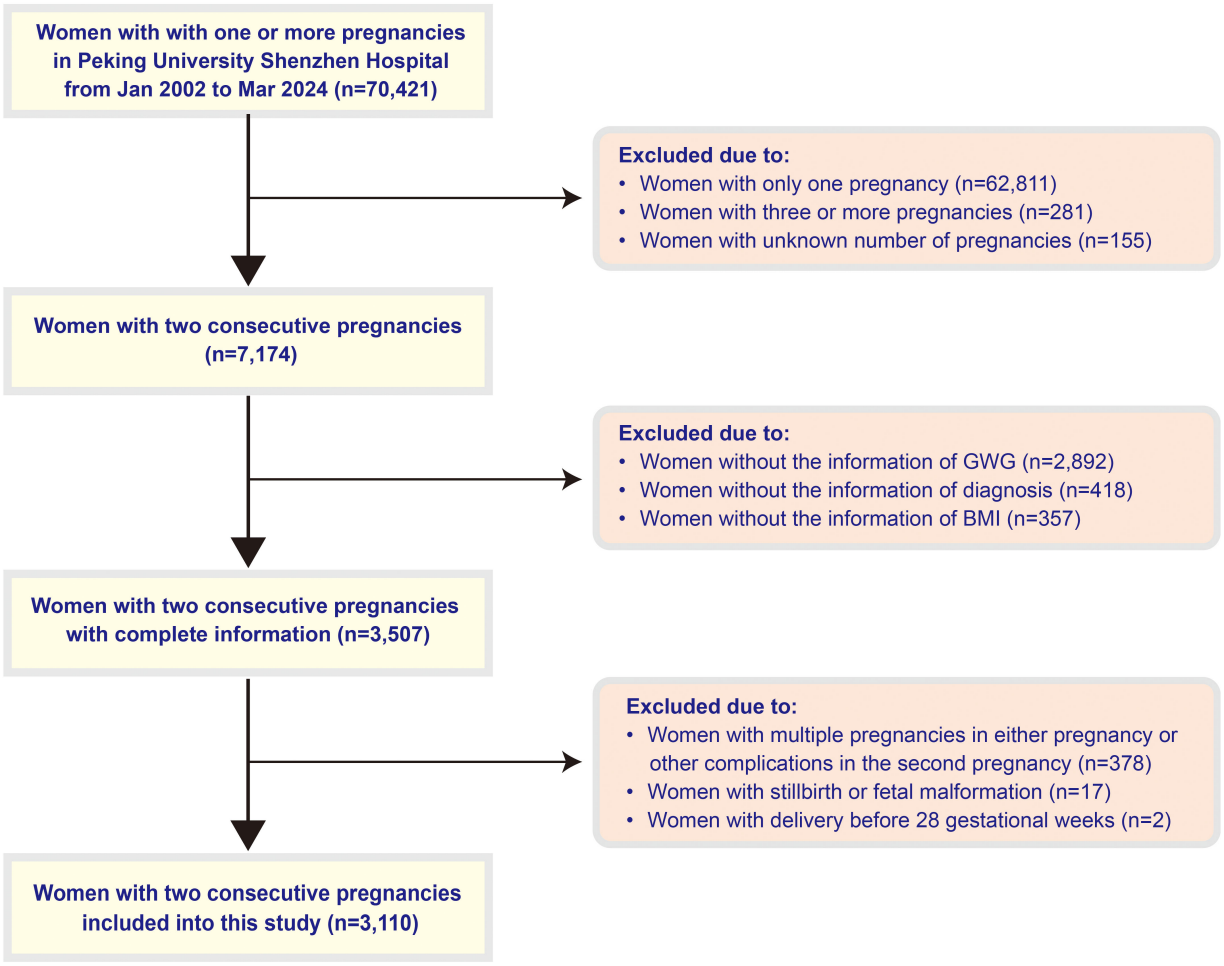
Flow chart showing inclusion and exclusion in this study. BMI, body mass index; GWG, gestational weight gain.

This study included 3,110 women with consecutive singleton deliveries (**Figure 1**). Significant inter-pregnancy changes were observed in clinical parameters (**Supplementary Table 1**): Maternal age increased from 28.22 ± 3.17 to 32.55 ± 3.70 years (*P <*0.001), with pre-pregnancy BMI rising from 20.47 ± 2.56 to 21.36 ± 2.84 kg/m² (*P <*0.001). The incidence of GDM increased from 10.1% (316/3110) to 15.9% (496/3110) (*P <*0.001), accompanied by elevated cesarean section (CS) rates (39.5% *vs* 35%) and MAC proportions (5.5% *vs* 4.4%) (both *P <*0.05). Weight categories showed polarization trends: underweight (UW) prevalence decreased (13.6% *vs* 21.8%) while overweight/obesity (OB) increased (16.3% *vs* 8.9%) (both *P <*0.001). The proportion of younger mothers (YMA) (<35 years) significantly declined from 96.9% to 70.8% (*P <*0.001).

### 3.2 Differences in f-BW and other risk factors between s-GDM and s-ND groups

Significant differences were observed between the s-GDM and s-ND groups (**Table 1**). The s-GDM group had significantly higher levels of s-MA, s-BMI, IPI, and IPWC (all P < 0.05), while its s-GWG was significantly lower (P < 0.05) when compared with the s-ND group The median interpregnancy interval (IPI) was 51.35 months in the subsequent GDM (s-GDM) group compared with 44.12 months in the subsequent non-GDM (s-ND) group, with a median difference of 7.23 months. The median interpregnancy weight change (IPWC) was 1.17 kg/m² in the s-GDM group versus 0.78 kg/m² in the s-ND group, with a median difference of 0.39 kg/m². For categorical outcomes, the s-GDM group showed elevated rates of f-MAC, f-LGA, f-GDM, f-CS, and extreme birth weight percentiles in the first pregnancy (>97th) (*P* < 0.05). Conversely, small-for-gestational-age infants (s-SGA) and lower birth weight percentiles in the first pregnancy (3^rd^–10^th^ percentile) were less frequent in the s-GDM group (*P* < 0.05). No significant differences were found in neonatal sex, hypertensive disorders, intrahepatic cholestasis, or other birth weight categories in the first pregnancy (*P* > 0.05) (**Table 1**).

**Table 1:** Comparison of potential risk factors between s-GDM and s-ND group.

Potential risk factors	s-GDM (n=496)	s-ND (n=2614)	*P*
continuous variables (x ± s, median[Q1-Q3])			
f-BW (g)	3270.79 ± 481.28	3239.07 ± 422.01	0.134
f-GWD (weeks)	**38.62 ± 1.539**	**38.92 ± 1.381**	**<0.001**
IPI (months)	**51.35 (36.46-75.73)**	**44.12 (29.63-65.37)**	**<0.001**
IPWC (kg/m^2^)	**1.17 (0.17-2.36)**	**0.78 (-0.1-1.84)**	**0.001**
s-MA (years)	**33.77 ± 3.537**	**32.32 ± 3.678**	**<0.001**
s-BMI (kg/m^2^)	**21.89 (20.06-24.03)**	**20.82 (19.22-22.74)**	**<0.001**
s-GWG (kg)	**11.92 ± 4.197**	**13.50 ± 2.226**	**<0.001**
categorical variables [n (%)]			
Categories of f-BW			
C1: <2500g	21(4.23)	85(3.25)	0.269
C2: 2500g≤ to 3000g	94(18.95)	557(21.31)	0.237
C3: 3000g≤ to 3500g	218(43.95)	1241(47.48)	0.149
C4: 3500g≤ to 4000g	131(26.41)	626(23.95)	0.241
C5: ≥ 4000g (f-MAC)	**32(6.45)**	**105(4.02)**	**0.023**
Categories of f-BW percentile			
P1: <3^rd^	5(1.01)	30(1.15)	0.787
P2: 3^rd^≤ to 10^th^	**22(4.44)**	**199(7.61)**	**0.012**
P3: 10^th^≤ to 25^th^	60(12.10)	396(15.15)	0.078
P4: 25^th^≤ to 50^th^	121(24.40)	694(26.55)	0.317
P5: 50^th^≤ to 75^th^	128(25.81)	613(23.45)	0.259
P6: 75^th^≤ to 90^th^	86(17.34)	442(16.91)	0.815
P7: 90^th^≤ to 97^th^	42(8.47)	174(6.66)	0.146
P8: ≥ 97^th^	**32(6.45)**	**66(2.52)**	**<0.001**
Han nationality	481 (96.98)	2506 (95.87)	0.246
f-LGA (P7+P8)	**74(14.91)**	**239(9.41)**	**<0.001**
f-SGA (P1+P2)	**27 (5.44)**	**229 (8.76)**	**0.014**
f-GDM	**177(35.69)**	**139(5.31)**	**<0.001**
f-CS	**220 (44.35)**	**871 (32.98)**	**<0.001**
f-MNB	248(50.00)	1284(49.12)	0.719
f-ICP	1(0.201)	14(0.536)	0.325
f-HDP	8 (1.612)	32 (1.224)	0.281

f-, in the first pregnancy; s-, in the second pregnancy; GDM, gestational diabetes mellitus; ND, no diabetes; BW, birth weight; GWD, gestational week at delivery; IPI, interpregnancy interval; IPWC, interpregnancy BMI change; MA, maternal age; BMI, body mass index; GWG, gestational weight gain; MAC, macrosomia; SGA, small-for-gestational-age; LGA, large-for-gestational-age; CS, cesarean section; MNB, male newborn; ICP, intrahepatic cholestasis of pregnancy; HDP, hypertensive disorders of pregnancy. Bold values indicate P < 0.05.

### 3.3 The association between f-BW and s-GDM in RCS analyses

**Figure 2:**
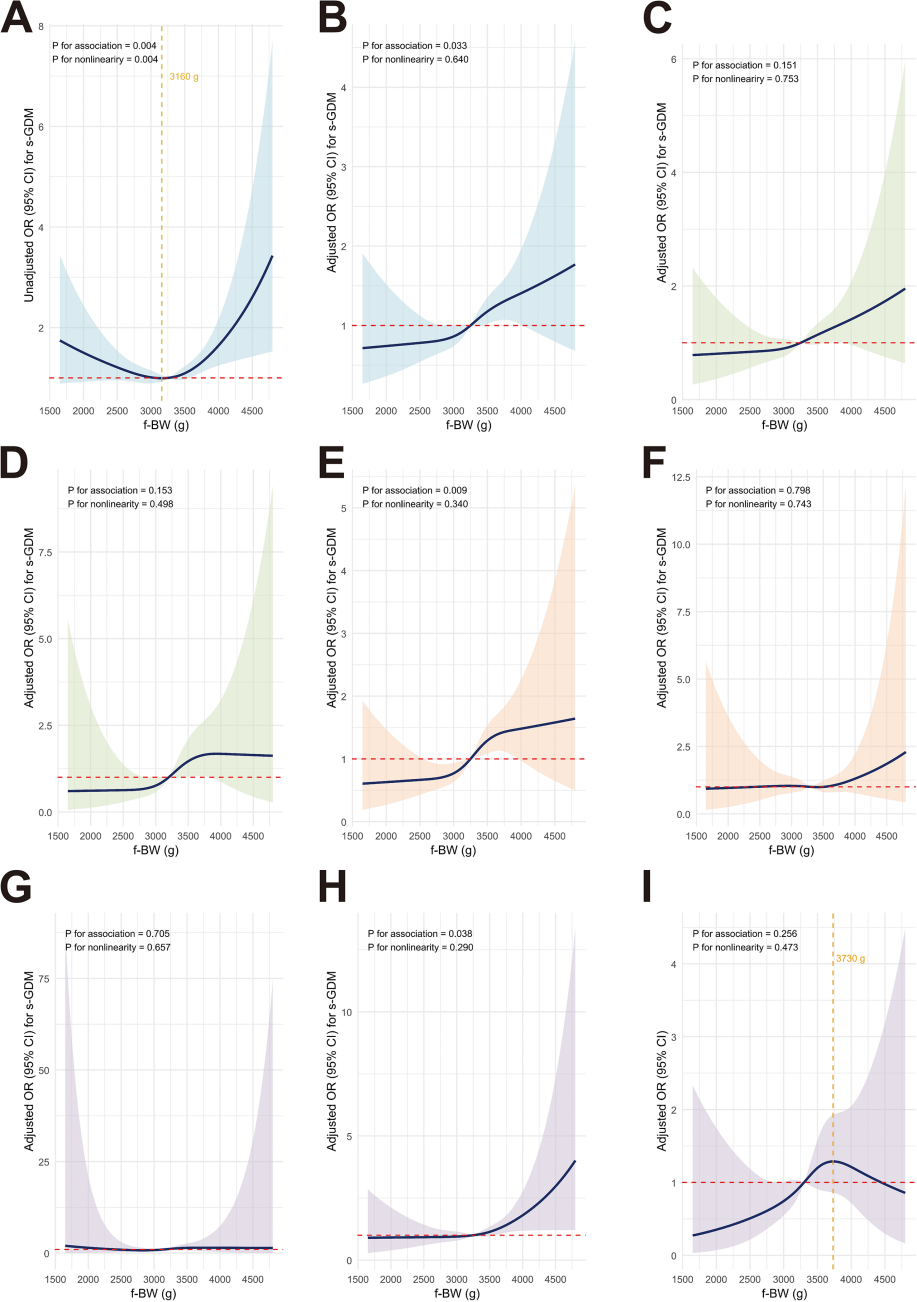
Associations between f-BW and s-GDM evaluated by RCS analyses s-GDM, gestational diabetes mellitus in the second pregnancy; f-BW, the neonatal birth weight in the first pregnancy; **(A)** shows unadjusted data, while **(B)** presents data adjusted for f-GDM, f-CS, f-GWD, IPWC, IPI, s-BMI, s-MA and s-GWG in the overall population; **(C-I)** display adjusted data for subgroups stratified by f-ND **(C)**, f-GDM **(D)**, s-YMA **(E)**, s-AMA **(F)**, s-UW **(G)**, s-NW **(H)**, and s-OB **(I)**. The reference group is the median of s-BW (3250g). Solid lines and shaded areas represent ORs and 95% CIs, respectively. Red horizontal dashed lines represented the OR value equal to 1.

The unadjusted RCS model demonstrated a J-shaped curve for the significant association between f-BW and s-GDM (P for association=0.004) (**Figure 2A**). However, after adjusting for f-GDM, f-CS, f-GWD, IPI, IPWC, s-BMI, s-MA, and s-GWG, the J-shaped curve disappeared though the association remained borderline significant (**Figure 2B**, P for association = 0.033) with nonsignificant nonlinearity (P for non-linearity = 0.640). The upper tail of f-BW (> 3500 g) in the overall population did not consistently have odds ratios (ORs) above 1 (95% confidence interval (CI) crossed the null line), indicating a nonsignificant dose-response pattern.

Among subgroup analyses (**Figures 2C–I**), adjusted RCS models only confirmed a significant positive association between f-BW and s-GDM in subgroups of s-YMA (**Figure 2E**) and s-NW (**Figure 2H**) (P for association < 0.001), with nonsignificant nonlinearity (P for non-linearity > 0.05). Specifically, subgroup of f-NW (**Figure 2H**) demonstrated an even stronger dose-response pattern in the upper tail (> 3500 g), where ORs and CIs consistently exceeded OR = 1, reinforcing the dose-dependent risk amplification in these high-exposure categories. In other subgroups (**Figures 2C, D, F, G, I**), neither the associations were nonsignificant (P for association > 0.05) nor the upper tail ORs did not consistently stay above 1 (the upper tail 95% CI crossed 1, which limited the interpretation of dose - response).

### 3.4 The association between f-BW percentile categories and s-GDM in RCS analyses

**Figure 3:**
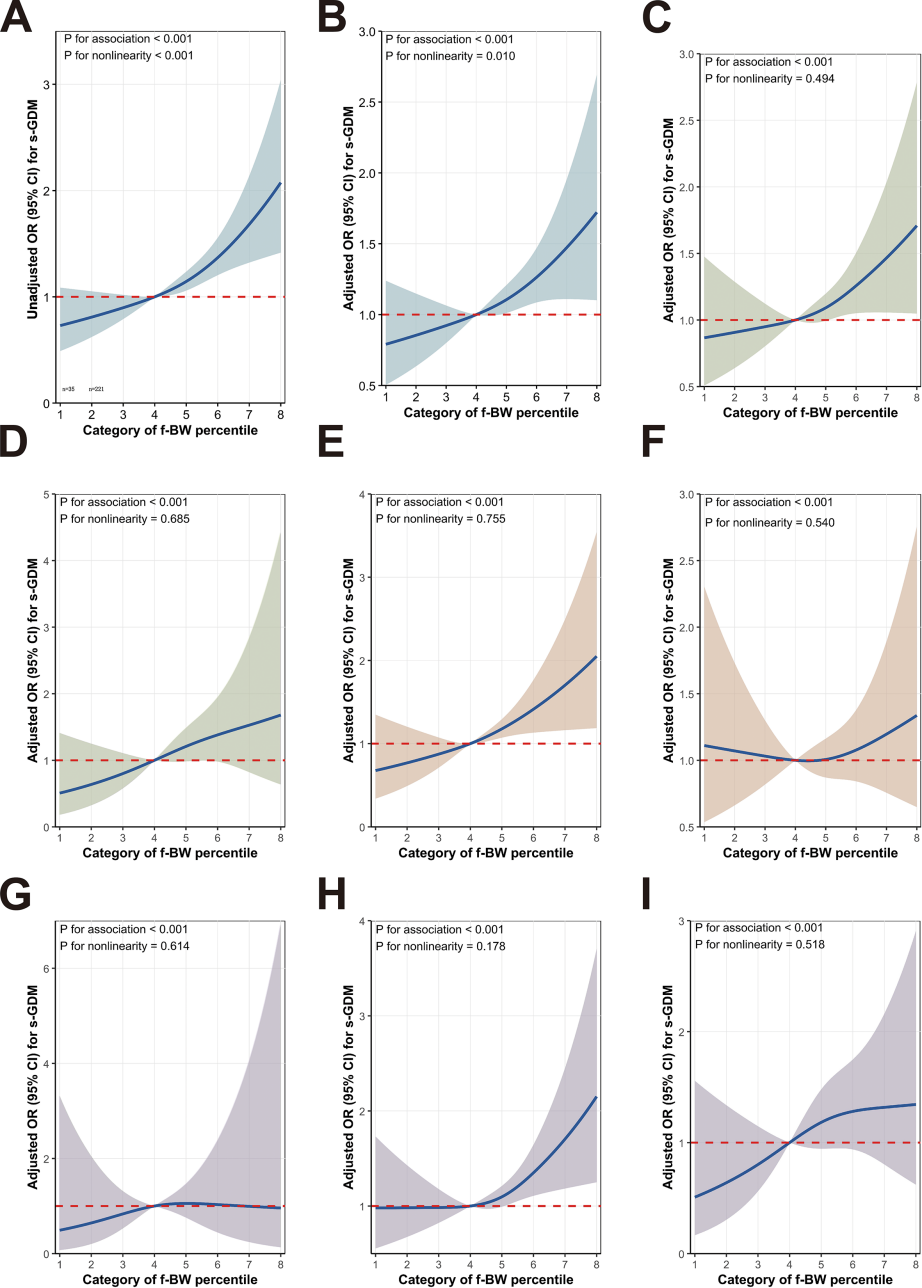
Associations between the category of f-BW percentiles and s-GDM evaluated by RCS analyses s-GDM, gestational diabetes mellitus in the second pregnancy; f-BW, the neonatal birth weight in the first pregnancy; **(A)** shows unadjusted data, while **(B)** presents data adjusted for f-GDM, f-CS, f-GWD, IPWC, IPI, s-BMI, s-MA and s-GWG in the overall population; **(C-I)** display adjusted data for subgroups stratified by f-ND **(C)**, f-GDM **(C)**, s-YMA **(E)**, s-AMA **(F)**, s-UW **(G)**, s-NW **(H)**, and s-OB **(I)**. Category of f-BW percentile is based on the 2020 China standards, P1: <3 ^rd^ (n=35); P2: 3^rd^≤ to 10^th^ (n=221); P3: 10^th^≤ to 25^th^ (n=456); P4: 25^th^≤ to 50^th^ (n=815); P5: 50^th^≤ to 75^th^ (n=741); P6: 75^th^≤ to 90^th^ (n=528); P7: 90^th^≤ to 97^th^ (n=216); P8: ≥ 97^th^ (n=98). The reference group is P4 (25th-50th percentiles). Solid lines and shaded areas represent ORs and 95% CIs, respectively. Red horizontal dashed lines represented the OR value equal to 1.

In the unadjusted RCS model (**Figure 3A**), f-BW percentiles showed a significant upward trend in s-GDM risk (P for association < 0.001). After adjusting for f-GDM, f-CS, f-GWD, IPI, IPWC, s-BMI, s-MA, and s-GWG, this association remained significant (**Figure 3B**, P for association < 0.001) with a significant nonlinearity (P for non-linearity = 0.010). Notably, the upper tail categories (P6–P8) in the overall population exhibited a pronounced dose-response relationship, with odds ratios (ORs) and 95% CIs steadily rising above the null line (OR = 1), indicating a significant dose-dependent risk elevation.

Across all subgroups (**Figures 3C–I**), adjusted RCS models confirmed a significant positive association between f-BW percentiles and s-GDM (P for association < 0.001), with nonsignificant nonlinearity (P for non-linearity > 0.05). Specifically, subgroups of f-ND (**Figure 3C**), s-YMA (**Figure 3E**), and s-NW (**Figure 3H**) demonstrated an even stronger dose-response pattern in the upper tail (P6–P8), where ORs and CIs consistently exceeded OR = 1, reinforcing the dose-dependent risk amplification in these high-exposure categories.

### 3.5 The association of f-LGA and f-MAC with s-GDM in univariate and multivariable regression

In unadjusted analyses (Model 1), the highest fetal weight category (C5: ≥4000g), the highest percentile category (P7+P8: ≥90th), f-MAC, and f-LGA were all significantly associated with increased s-GDM risk compared to their respective reference groups (**Table 2**). After adjustment (Models 2 and 3), the association for the highest percentile category (P7+P8) remained significant (*P* < 0.05), while that for the highest fetal weight category (C5: ≥4000g) became non-significant (*P* > 0.05). Lower and intermediate weight categories (C1, C2, C4; P1+P2, P3, P5, P6) showed no significant associations in any model (*P* > 0.05). Significant positive trends were observed across percentile categories in all models (*P*-trend < 0.05), but not across categorical weight groups (*P*-trend ≥ 0.05). In the analysis of binary variables after adjustment (Model 2 and Model 3), only f-LGA retained significance (*P* < 0.05), whereas f-MAC was no longer significant (*P* > 0.05).

**Table 2:** Analyses of the association of f-LGA and f-MAC with s-GDM across unadjusted and adjusted models.

Independent variable	s-GDM Cases (n)	Model 1		Model 2		Model 3	
		OR (95% CI)	*P* for trend	aOR (95% CI)	*P* for trend	aOR (95% CI)	*P* for trend
categories of f-BW							
C1 (n=106)	21	1.406 (0.834-2.274)	0.050	1.480 (0.873-2.407)	0.300	0.728 (0.349-1.462)	0.310
C2 (n=651)	94	0.961 (0.737-1.244)		0.976 (0.747-1.268)		0.751 (0.546-1.026)	
C3 (n=1459)	218	1.000		1.000		1.000	
C4 (n=757)	131	1.191 (0.938-1.508)		1.102 (0.865-1.399)		1.243 (0.942-1.635)	
C5 (n=137)	32	**1.735 (1.123-2.615)**		1.354 (0.865-2.067)		1.342 (0.800-2.197)	
categories of f-BW percentile							
P1+P2 (n=256)	27	0.676 (0.427-1.038)	**<0.001**	0.720 (0.453-1.109)	**0.021**	0.904 (0.554-1.435)	**0.025**
P3 (n=456)	60	0.869 (0.620-1.208)		0.910 (0.647-1.269)		0.824 (0.557-1.206)	
P4 (n=815)	121	1.000		1.000		1.000	
P5 (n=741)	128	1.198 (0.913-1.572)		1.156 (0.879-1.521)		1.200 (0.879-1.638)	
P6 (n=528)	86	1.116 (0.824-1.506)		1.081 (0.797-1.463)		1.160 (0.824-1.630)	
P7+P8 (n=314)	74	**1.768 (1.275-2.441)**		**1.464 (1.046-2.037)**		**1.536 (1.044-2.248)**	
binary variable analysis							
f-MAC (n=137)	32	**1.609(1.064-2.432)**	/	1.279(0.835-1.958)	/	1.271(0.782-2.067)	/
f-LGA (n=314)	74	**1.743(1.316-2.307)**		**1.438(1.077-1.921)**		**1.458(1.045-2.035)**	

f-, in the first pregnancy; s-, in the second pregnancy; GDM, gestational diabetes mellitus; MAC, macrosomia; LGA, large-for-gestational-age; BW, the neonatal birth weight; C1: <2500g; C2: 2500g≤ to 3000g; C3: 3000g≤ to 3500g; C4: 3500g≤ to 4000g; C5: ≥ 4000g. Category of f-BW percentile is based on the 2020 China standards, P1: <3 ^rd^; P2: 3^rd^≤ to 10^th^; P3: 10^th^≤ to 25^th^; P4: 25^th^≤ to 50^th^; P5: 50^th^≤ to 75^th^; P6: 75^th^≤ to 90^th^; P7: 90^th^≤ to 97^th^; P8: ≥ 97^th^; Categories P1+P2 (<10^th^ percentile) and P7+P8 (≥90^th^ percentile) were combined due to low sample sizes. OR, odds ratio; CI, confidence interval; Model 1: unadjusted; Model 2: adjusted for s-BMI; Model 3: adjusted for f-GDM, f-CS, f-GWD, IPI, IPWC, s-BMI, s-MA, and s-GWG. Bold values indicate P <0.05.

### 3.6 The association of f-MAC with s-GDM in stratified analyses

In stratified analyses, Model 1 (unadjusted) demonstrated a significant association between f-MAC and s-GDM in the s-YMA subgroup, whereas no significant associations were observed in the f-ND, f-GDM, s-AMA, s-UW, s-NW, or s-OB subgroups. Model 2 (adjusted for s-BMI) retained a similar positive trend in the s-YMA subgroup, but this trend was attenuated. These patterns aligned with the results of Model 3 (fully adjusted for f-GDM, f-CS, f-GWD, IPI, IPWC, s-BMI, s-MA, and s-GWG), where no significant associations between f-MAC and s-GDM persisted across any subgroups (**Supplementary Table 2**). Formal interaction tests revealed that only maternal age significantly modified the f-MAC-s-GDM association (P for interaction = 0.042), while no significant effect modification was observed for prior GDM status (P for interaction = 0.606) or pre-pregnancy BMI (P for interaction = 0.645) (**Supplementary Table 2**).

### 3.7 The association of f-LGA with s-GDM in stratified analyses

Stratified analyses revealed heterogeneous associations between f-LGA and s-GDM across subgroups (**Supplementary Table 3**): Model 1 (unadjusted) and Model 2 (adjusted for s-BMI) both showed significant positive associations between f-LGA and s-GDM in the f-ND, s-YMA, and s-NW subgroups, while no significant associations were noted in the f-GDM, s-AMA, s-UW, or s-OB subgroups—aligning with the results of Model 3 (fully adjusted for f-GDM, f-CS, f-GWD, IPI, IPWC, s-BMI, s-MA, and s-GWG). In Model 3, the significant associations persisted in the f-ND (aOR=1.544, 95% CI:1.069–2.232), s-YMA (aOR=1.668, 95% CI:1.095–2.542), and s-NW (aOR=1.757, 95% CI:1.169–2.641) subgroups. Conversely, no significant associations were detected in the f-GDM (aOR=1.148, 95% CI:0.547–2.412), s-AMA (aOR=1.206, 95% CI:0.697–2.086), s-UW (aOR=0.584, 95% CI:0.059–5.768), or s-OB (aOR=1.208, 95% CI:0.673–2.170) subgroups. Formal interaction tests confirmed that prior GDM status, maternal age, and pre-pregnancy BMI all significantly modified the f-LGA-s-GDM association, with a consistent *P* for interaction of <0.001 across all three stratified factors (**Supplementary table 3**).

## 4 Discussion

This study provides robust evidence that delivering a LGA infant in the first pregnancy is significantly associated with the risk of GDM in a subsequent pregnancy, particularly among women without prior GDM, younger mothers (<35 years), and those with normal pre-pregnancy BMI. In contrast, macrosomia (MAC) alone did not demonstrate an independent association after adjusting for metabolic and obstetric confounders. These findings highlight the distinct roles of fetal overgrowth phenotypes in association with maternal metabolic health, with implications for risk stratification and clinical management.

The observed association between f-LGA and s-GDM aligns with a large Israeli case-control study (n=47,823) in women without prior GDM, which reported a 70% increased risk of subsequent GDM (aOR=1.7, 95% CI:1.1–2.5) (30). LGA, which is a phenotype reflecting fetal overgrowth relative to population-specific norms, may serve as a proxy for persistent maternal metabolic dysfunction, including insulin resistance (41), dyslipidemia such as elevated apolipoprotein levels (42), serum total cholesterol (43), and subclinical glucose intolerance that persists across pregnancies. Metabolomic studies further link LGA to increased maternal carnitines, steroid hormones, and lipid metabolites in late pregnancy (44), all of which are implicated in the pathophysiology of GDM, such as insulin resistance and carbohydrate-lipid metabolic dysregulation (28). These metabolic mechanisms likely persist interpregnancy and specifically predispose to GDM via a ‘β-cell reserve depletion’ pathway: In the first pregnancy, insulin resistance and dyslipidemia reduce maternal β-cell reserve. In the subsequent pregnancy, physiological insulin demand increases (41); women with depleted β-cell reserve cannot compensate, leading to glucose intolerance and s-GDM. Long-term follow-up data reinforce this association: women without prior GDM who delivered LGA infants face a higher risk of developing prediabetes and type 2 diabetes 10–14 years post-delivery (31, 32), underscoring LGA’s role as a marker of enduring metabolic perturbation.

Stratified analyses (**Supplementary Table 3**) and restricted cubic spline (RCS) modeling (**Figures 3C–I**) collectively demonstrate that the association between f-LGA and s-GDM is strongly modulated by maternal metabolic risk factors. In low-risk subgroups, specifically women without prior GDM (f-ND), younger mothers (s-YMA, <35 years), and those with normal pre-pregnancy BMI (s-NW), f-LGA was significantly associated with s-GDM. RCS curves in these subgroups exhibited clear upward dose-response trends at higher birth weight percentiles (≥90th), which may raise the hypothesis that among individuals without preexisting metabolic abnormalities (free of preexisting hyperglycemia, advanced age, or obesity), f-LGA could serve as a potential marker of intrinsic insulin resistance or genetic predisposition to glucose dysregulation. This observation aligns with genetic studies linking LGA risk to maternal-fetal polygenic variants associated with birth weight and insulin sensitivity (27, 45), which hypothetically could persist interpregnancy to increase GDM susceptibility.

Conversely, logistic regression and RCS analyses revealed attenuated or non-significant associations in high-risk subgroups (prior GDM [f-GDM], advanced maternal age [s-AMA, ≥35 years], or overweight/obesity [s-OB]). The ‘masking’ hypothesis explains the null f-LGA-s-GDM association in high-risk subgroups: In women with prior GDM, gestational hyperglycemia itself is a dominant driver of fetal overgrowth (30) and recurrent GDM (17), making the additional ‘signal’ of prior LGA negligible. Notably, while f-LGA was not associated with recurrent GDM here, women with prior GDM and LGA delivery face elevated long-term metabolic syndrome risk (46), underscoring the need for postpartum surveillance beyond GDM screening. In women with advanced age (≥35 years), age-related β-cell decline (13) is the primary GDM risk factor, overshadowing LGA’s incremental effect. In overweight/obese women, obesity-induced insulin resistance (14) is so pronounced that LGA no longer adds meaningful predictive value. In contrast, LGA remains a strong marker in normal BMI women because these women lack obesity-related insulin resistance, thus LGA acts as a salient indicator of underlying metabolic perturbations, hypothesized to include intrinsic insulin resistance and genetic predisposition, that would otherwise be unrecognized in a low-risk population. Corresponding RCS curves showed flat trends with upper-tail odds ratios crossing 1, supporting the hypothesis that preexisting metabolic stressors mask the associative value of f-LGA—consistent with research showing maternal BMI and prior LGA more strongly influence fetal growth in normoglycemic women (47).

Significant interaction effects (P < 0.001 for age and BMI) confirm the contextual nature of the f-LGA/s-GDM association. In low-risk groups, f-LGA acts as a “signal” of underlying dysregulation, while in high-risk groups, it is overshadowed by established risk factor “noise.” This aligns with metabolomic studies linking LGA to maternal carnitine and lipid metabolite abnormalities (44), which are more likely to persist in women without prior metabolic insults. Clinically, these results advocate for targeted GDM screening in low-risk multiparous women with f-LGA history, particularly younger, normal-weight individuals without prior GDM. For high-risk subgroups, management should prioritize modifiable factors such as pre-pregnancy weight loss, glycemic control over fetal growth history alone, while acknowledging the long-term metabolic risks associated with LGA delivery after hyperglycemia (46). In summary, the convergence of stratified and RCS analyses establishes f-LGA as a context-dependent risk marker, with greatest utility in populations unconfounded by overt metabolic disease. These findings elucidate how fetal overgrowth phenotypes interact with maternal factors to influence GDM pathogenesis, providing a framework for personalized prenatal care that integrates immediate and long-term metabolic risk stratification.

In this study, the association between prior macrosomia (f-MAC) and subsequent GDM lost statistical significance after multivariable adjustment (Model 3: aOR=1.271, 95% CI: 0.782–2.067), consistent with a Chinese multicenter study (aOR=1.64, 95% CI:0.95–2.83) that similarly found no independent association after adjusting for age, BMI, and interpregnancy factors (34). However, this contrasts with meta-analyses reporting prior macrosomia as a risk factor for recurrent GDM (OR = 3.48–4.41) (17, 18) and a NHANES cohort showing a 21% increased risk of T2DM after macrosomia delivery (33). These discrepancies likely stem from methodological heterogeneity, including population diversity and confounder adjustment. For example, the Israeli study (assessing non-diabetic women with previous macrosomic infants) found a significant association between first-pregnancy macrosomia (f-MAC, ≥4000 g) and subsequent GDM (s-GDM) (aOR=1.9, 95% CI:1.1–3.4) (30), which differs from our findings, and two key factors explain this divergence: (1) Adjustment for interpregnancy weight change (IPWC): The Israeli study’s multivariate models adjusted for confounders (maternal age, parity, previous miscarriage/cesarean, BMI, interpregnancy interval [IPI]) but not IPWC. In our data, unadjusted models showed a significant f-MAC and s-GDM association (OR = 1.609, 95% CI:1.064-2.432), which attenuated to non-significance after IPWC adjustment, indicating IPWC explains much of the macrosomia effect in Chinese women, a factor unaddressed in the Israeli analysis. (2) Population-specific birth weight distribution: The Israeli study reported higher mean previous birth weights (3229.9 ± 488.2 g [non-GDM] *vs*. 3336.9 ± 587.4 g [GDM]), with macrosomia occurring in 9.6% of women with s-GDM (30). In contrast, our Chinese cohort had a lower mean previous birth weight (3250 g), and macrosomia was rare (4.4% - 5.5% of the total cohort). This means the ≥4000 g threshold is more specific to metabolic dysfunction in Israeli women (reflecting a larger deviation from their average birth weight) but less specific in Chinese women (an extreme, metabolically less meaningful cutoff), weakening its association with s-GDM in our study.

The divergent roles of LGA and macrosomia in association with subsequent GDM stem from their fundamental definitional differences. LGA reflects relative fetal overgrowth within population-specific, sex- and gestational age-adjusted norms (26), making it a more biologically nuanced marker of maternal-fetal metabolic dysregulation. In contrast, macrosomia’s absolute threshold (≥4000 g) lacks contextual growth reference, potentially conflating constitutional fetal size with pathological overgrowth. This is supported by genetic studies showing LGA risk is driven by maternal-fetal polygenic variants associated with birth weight and glucose metabolism (27, 45), whereas macrosomia often correlates with modifiable factors like excessive gestational weight gain (22). Mechanistically, LGA’s stronger link to maternal insulin resistance (41) and dyslipidemia (42, 43), evinced by HbA1c associations with LGA but not macrosomia in both GDM (pooled RR = 1.70, P <0.001) (48) and non-GDM populations (aOR=1.47 *vs*. 1.12) (49), underscores its role as a marker of persistent metabolic dysfunction. In summary, the lack of independent association for macrosomia highlights the limitations of absolute weight thresholds in assessing GDM risk, while LGA’s percentile-based definition provides a more robust proxy for underlying maternal metabolic health. These findings advocate for routine integration of fetal growth percentiles, rather than sole reliance on macrosomia criteria, in postpartum counseling and subsequent pregnancy risk assessment.

The strengths of this study include a large, well-phenotyped cohort (n=3,110) with consecutive singleton deliveries spanning 22 years, standardized LGA definition using China-specific 2020 growth standards, and rigorous statistical analyses (restricted cubic splines for non-linear trends, multivariable logistic regression with sequential adjustment, and stratified analyses for effect modification). Limitations include the single-center, retrospective design, which limits causal inference and generalizability to rural or non-tertiary settings. Regional clinical practices specific to our urban tertiary hospital (e.g., uniform prenatal care protocols, low preterm birth rates) may enhance internal validity but restrict external applicability to populations with different healthcare access or practice patterns. A notable limitation is the lack of data on important confounders, including maternal dietary patterns, physical activity levels, and family history of diabetes. These factors are known to influence both fetal growth (LGA/MAC) and GDM risk, and their exclusion may introduce residual confounding that could slightly overestimate or underestimate the observed association between f-LGA and s-GDM. Additionally, while we harmonized GDM diagnostic criteria across the study period (pre-2010 two-step *vs*. post-2010 IADPSG one-step method), potential residual bias from diagnostic protocol changes cannot be fully ruled out.

Collectively, a history of LGA delivery is independently associated with subsequent GDM, particularly in metabolically vulnerable subgroups without preexisting obesity or dysglycemia. These findings support incorporating LGA history into risk-stratified screening protocols for multiparous women—especially younger, normal-weight individuals without prior GDM. Such integration could enhance early detection of high-risk pregnancies, facilitating timely preventive interventions. While macrosomia showed limited associative relevance for direct GDM risk stratification, it may signal opportunities for mitigating modifiable risks through weight management. Future prospective studies should: (1) validate LGA as a clinical predictor of s-GDM in multicenter or prospective cohorts—a critical step to enhance generalizability beyond our single-center sample, especially in rural or non-tertiary care settings; (2) elucidate underlying mechanisms by integrating biomarker data (e.g., insulin resistance markers, placental dysfunction-related factors, lipid metabolites) or genetic data (e.g., maternal-fetal polygenic variants linked to glucose metabolism), alongside previously proposed pathways like placental dysfunction or epigenetic programming; (3) assess preventive strategies (e.g., preconception weight optimization, metformin prophylaxis) in high-risk women with a history of LGA birth. Additionally, multi-ethnic cohorts are further warranted to establish generalizability across diverse populations, complementing the multicenter validation effort.

## Data Availability

The raw data supporting the conclusions of this article will be made available by the authors, without undue reservation.
