# Supplementary material for: History of large-for-gestational-age birth is independently associated with subsequent gestational diabetes in Chinese multiparous women: a retrospective cohort study
Source: Front Endocrinol (Lausanne). 2025 Dec 4;16:1678319. doi: 10.3389/fendo.2025.1678319 (PMC12711537; doi:10.3389/fendo.2025.1678319)
Supplement: Supplementary file 1 [file Table1.docx]

Supplementary Table 1 Comparison of parameters between the first and the second pregnancies

|  | The first pregnancies  (n=3110) | The second pregnancies  (n=3110) | t/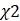 | *P* |
| --- | --- | --- | --- | --- |
| MA (years, 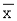±s) | 28.22±3.17 | 32.55±3.70 | 99.267 | ＜0.001 |
| BMI (kg/m^2^, 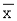±s) | 20.47±2.56 | 21.36±2.84 | 27.549 | ＜0.001 |
| GDM [n(%)] | 316（10.1%） | 496（15.9%） | 8.507 | ＜0.001 |
| CS [n(%)] | 1091（35%） | 1231（39.5%） | 9.666 | ＜0.001 |
| MAC[n(%)] | 137（4.4%） | 174（5.5%） | 4.634 | 0.031 |
| MN[n(%)] | 1532（49.2%） | 1679（53.9%） | 3.640 | ＜0.001 |
| UW[n(%)] | 679（21.8%） | 423（13.6%） | 5.482 | ＜0.001 |
| NW [n(%)] | 2152（69.2%） | 2179（70.1%） | 20.677 | ＜0.001 |
| OB [n(%)] | 279（8.9%） | 508（16.3%） | 6.669 | ＜0.001 |
| YMA[n(%)] | 3014（96.9%） | 2202（70.8%） | 95.721 | ＜0.001 |

MA, maternal age; BMI, body mass index; GDM, gestational diabetes mellitus; CS, caesarean section; MAC, macrosomia; MN, male newborn; UW, underweight; NW, normal weight; OB, overweight; YMA, young maternal age.
