# Supplementary material for: History of large-for-gestational-age birth is independently associated with subsequent gestational diabetes in Chinese multiparous women: a retrospective cohort study
Source: Front Endocrinol (Lausanne). 2025 Dec 4;16:1678319. doi: 10.3389/fendo.2025.1678319 (PMC12711537; doi:10.3389/fendo.2025.1678319)
Supplement: Supplementary file 2 [file Table2.docx]

**Supplementary Table 2 Stratified analyses of the association between f-MAC and s-GDM**

|  | Model 1 |  | Model 2 |  | Model 3 | P for interaction |
| --- | --- | --- | --- | --- | --- | --- |
|  | OR (95% CI) |  | aOR (95% CI) |  | aOR (95% CI) |  |
| f-ND (n=2794) | 1.498(0.893-2.515) |  | 1.238(0.729-2.100) |  | 1.308(0.750-2.279) | 0.606 |
| f-GDM (n=316) | 1.145(0.475-2.762) |  | 0.803(0.318-2.028) |  | 1.137(0.432-2.992) |  |
|  |  |  |  |  |  |  |
| s-YMA (n=2202) | **2.190(1.318-3.641)** |  | **1.774(1.054-2.986)** |  | 1.532(0.834-2.814) | **0.042** |
| s-AMA (n=908) | 0.885(0.434-1.805) |  | 0.715(0.341-1.495) |  | 0.923(0.406-2.101) |  |
|  |  |  |  |  |  |  |
| s-UW (n=423) | 1.963(0.229-16.847) |  | 1.950(0.227-16.753) |  | 1.374(0.091-19.864) | 0.645 |
| s-NW (n=2179) | 1.517(0.890-2.588) |  | 1.435(0.839-2.455) |  | 1.761 (0.965-3.213) |  |
| s-OB (n=508) | 1.151(0.573-2.315) |  | 1.061(0.522-2.157) |  | 0.933(0.418-2.082) |  |

f-, in the first pregnancy; s-, in the second pregnancy; GDM, gestational diabetes mellitus; MAC, macrosomia; ND, no diabetes; YMA, young maternal age (less than 35 years); AMA, advanced maternal age (35 years or more); UW, underweight; NW, normal weight; OB, overweight or obesity. Model 1: unadjusted; Model 2: adjusted for s-BMI; Model 3: adjusted for f-GDM, f-CS, f-GWD, IPI, IPWC, s-BMI, s-MA, and s-GWG. Bold values indicate P <0.05.
