# Supplementary material for: History of large-for-gestational-age birth is independently associated with subsequent gestational diabetes in Chinese multiparous women: a retrospective cohort study
Source: Front Endocrinol (Lausanne). 2025 Dec 4;16:1678319. doi: 10.3389/fendo.2025.1678319 (PMC12711537; doi:10.3389/fendo.2025.1678319)
Supplement: Supplementary file 3 [file Table3.docx]

**Supplementary Table 3 Stratified analyses of the association between f-LGA and s-GDM**

|  | Model 1 |  | Model 2 |  | Model 3 | P for interaction |
| --- | --- | --- | --- | --- | --- | --- |
|  | OR (95% CI) |  | aOR (95% CI) |  | aOR (95% CI) |  |
| f-ND（n=2794） | **1.710(1.215-2.406)** |  | **1.498(1.040-2.155)** |  | **1.544(1.069-2.232)** | ＜0.001 |
| f-GDM（n=316） | 1.349(0.705-2.579) |  | 1.087(0.522-2.263) |  | 1.148(0.547-2.412) |  |
|  |  |  |  |  |  |  |
| s-YMA(n=2202) | **2.143 (1.497-3.069)** |  | **1.564(1.030-2.376)** |  | **1.668(1.095-2.542)** | ＜0.001 |
| s-AMA(n=908) | 1.139(0.725-1.788) |  | 1.188(0.690-2.047) |  | 1.206(0.697-2.086) |  |
|  |  |  |  |  |  |  |
| s-UW(n=423) | 0.625 (0.081-4.827) |  | 0.577(0.056-5.525) |  | 0.584(0.059-5.768) | ＜0.001 |
| s-NW (n=2179) | **1.658 (1.164-2.361)** |  | **1.681(1.122-2.518)** |  | **1.757(1.169-2.641)** |  |
| s-OB (n=508) | 1.417 (0.860-2.335) |  | 1.341(0.808-2.225) |  | 1.208(0.673-2.170) |  |

f-, in the first pregnancy; s-, in the second pregnancy; GDM, gestational diabetes mellitus; LGA, large-for-gestational-age; ND, no diabetes; YMA, young maternal age (less than 35 years); AMA, advanced maternal age (35 years or more); UW, underweight; NW, normal weight; OB, overweight or obesity. Model 1: unadjusted; Model 2: adjusted for s-BMI; Model 3: adjusted for f-GDM, f-CS, f-GWD, IPI, IPWC, s-BMI, s-MA, and s-GWG. Bold values indicate P <0.05.
